# Ectopic Thyroid Carcinoma Presenting as a Superior Mediastinal Cystic Mass

**DOI:** 10.7759/cureus.94823

**Published:** 2025-10-17

**Authors:** Kei Kajihara, Takayuki Kawabata, Hiroyuki Koga, Kousuke Marutsuka, Takumi Okuda, Shinsuke Ide, Kuniyuki Takahashi

**Affiliations:** 1 Otolaryngology Head and Neck Surgery, Faculty of Medicine, University of Miyazaki, Miyazaki, JPN; 2 Otolaryngology Head and Neck Surgery, Miyazaki Prefectural Miyazaki Hospital, Miyazaki, JPN; 3 Pathology, Miyazaki Prefectural Miyazaki Hospital, Miyazaki, JPN

**Keywords:** cystic lesion, dysgenesis, ectopic thyroid, extragenesis, imaging, mediastinum, papillary carcinoma, surgery, thyroid cancer

## Abstract

Ectopic thyroids are thyroid tissue that develops in locations other than its normal position. During their developmental process, they rarely form outside the route of the thyroglossal duct, especially below the normal thyroid gland position. Most ectopic thyroids have normal histological findings, and malignant tumors are relatively rare. In this report, we present a case of ectopic thyroid carcinoma that presented as a mediastinal cystic lesion. A 46-year-old woman was incidentally diagnosed with a mediastinal cystic mass during a follow-up for another condition. The mass was surgically resected, and pathological examination revealed malignant cells in the small papillary area and positive staining of the superficial epithelium for thyroid transcription factor-1 using immunochemistry. An orthotopic normal thyroid gland without tumor lesions was observed on CT and MRI, leading to a diagnosis of papillary adenocarcinoma originating from an ectopic thyroid in the mediastinum. Although it must always be differentiated from microcarcinoma metastasis, ectopic thyroid carcinoma should be considered in the differential diagnosis of cystic lesions in the mediastinum. The follow-up and treatment strategies for ectopic thyroid carcinoma are controversial and require individualized consideration for each case.

## Introduction

Ectopic thyroids are thyroid tissue that develops in locations other than its normal position. During their developmental process, they rarely form outside the route of the thyroglossal duct, especially below the normal thyroid gland position [1,2]. They often form as lingual thyroids when the thyroid gland is absent from its normal position and sometimes occur in the lower part of the mentum or anterior neck during their developmental process [2-5]. However, other ectopic thyroids can also form as additional thyroid tissue in different locations, even when a normal thyroid gland is present [3]. As the histology of most ectopic thyroid tissues is typically normal, the development of malignant tumors is relatively rare, at approximately 1%-12%. [5-7]. In this report, we present a case of ectopic thyroid carcinoma that presented as a cystic lesion in the superior mediastinum.

## Case presentation

The patient was a 46-year-old woman who had undergone surgery for endometrial stromal sarcoma eight years ago. Follow-up whole-body computed tomography (CT) revealed a small mass in the superior mediastinum five years ago. For several years, the lesion did not change in size on CT. However, a CT taken one month ago revealed that the lesion had grown, leading to the patient visiting our department. The patient had no symptoms, including dyspnea or dysphagia. Physical examination, including visual inspection, palpation of the neck, and laryngoscopy, revealed no abnormalities. Blood examination showed calcium (Ca) 8.5 mg/dL, intact parathyroid hormone (PTH) 48 pg/mL, thyroid-stimulating hormone (TSH) 1.54 μU/mL, free thyroxine (FT4) 0.80 ng/dL, and free triiodothyronine (FT3) 2.31 pg/mL, with both parathyroid and thyroid function within normal limits.

**Figure 1:**
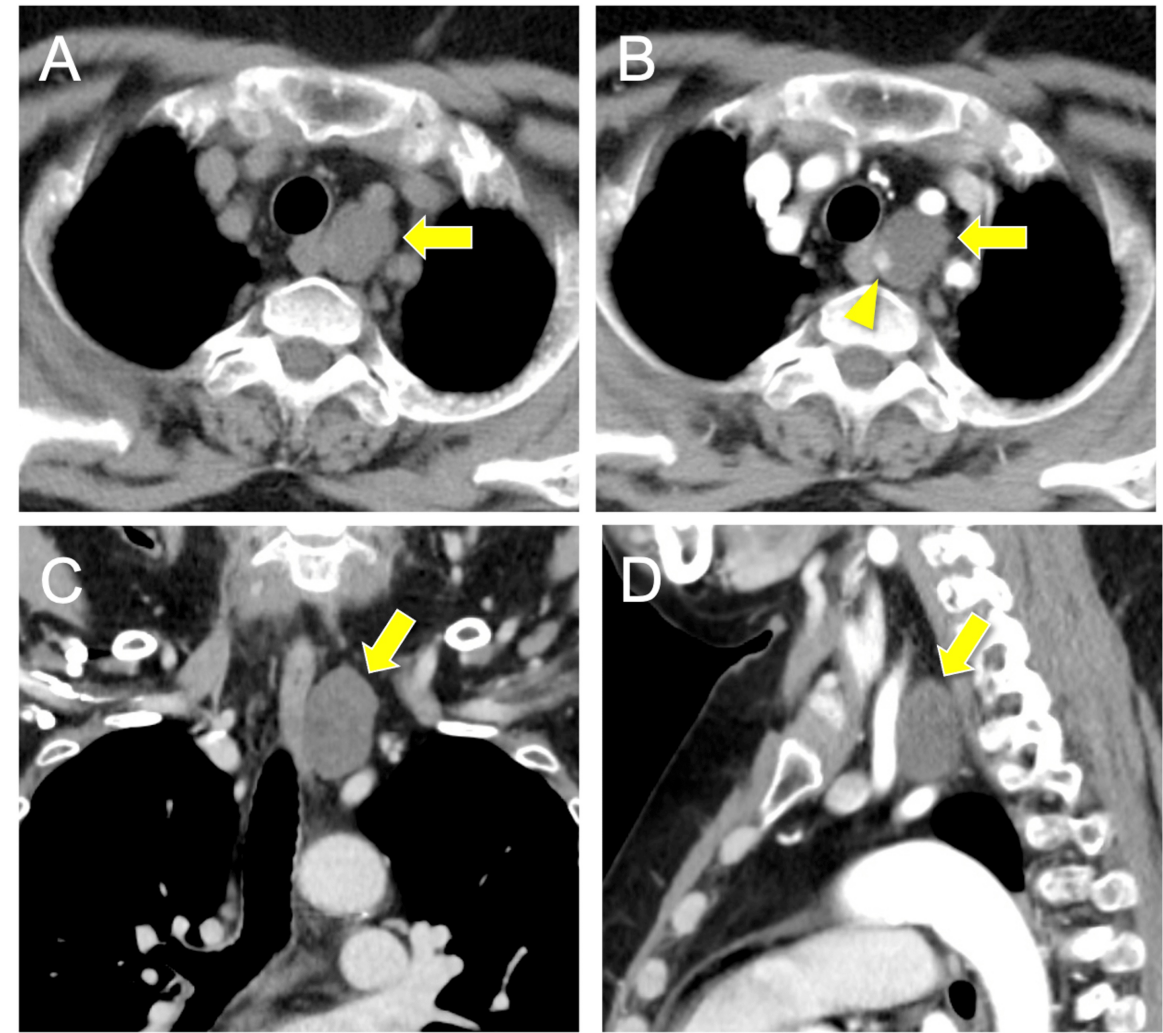
Preoperative CT images (A) A non-enhanced CT scan showed a mass in the superior mediastinum near the left side of the esophagus. (B) Contrast-enhanced CT showed that most of the mass was non-enhancing, but a small portion within the mass was contrast-enhancing (arrowhead). (C) Coronal section and (D) sagittal section of contrasted CT. The mass was located posterior to the common carotid artery and mediosuperior to the subclavian artery.

**Figure 2:**
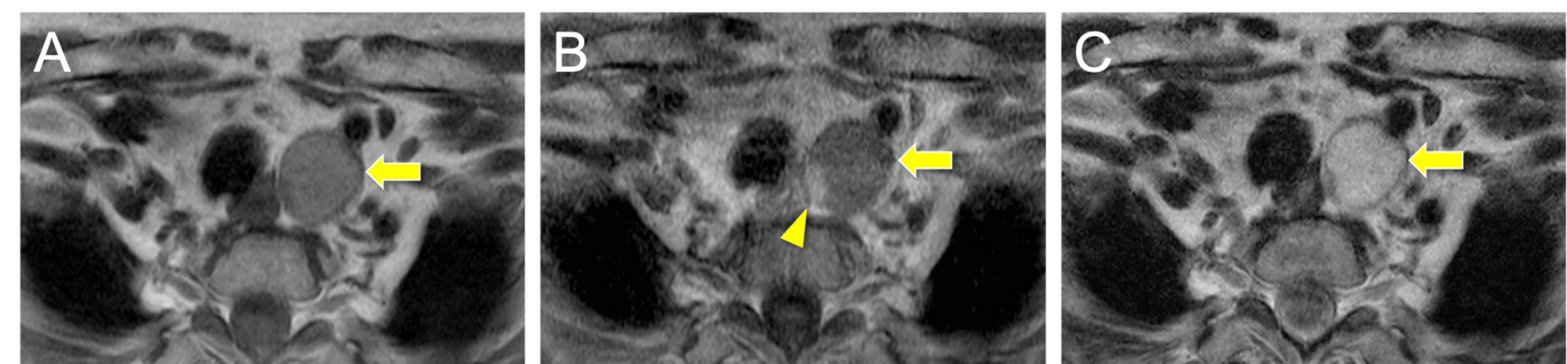
Preoperative MRI (A) The tumor showed hypointensity on T1-weighted MRI. (B) On gadolinium-enhanced MRI, only a small area corresponding to the CT-enhanced region showed enhancement (arrowhead). (C) The tumor showed hyperintensity on T2-weighted MRI images.

The CT revealed a mass in the superior mediastinum near the left side of the esophagus (Figure 1A). Most of the mass was non-enhancing, but a small portion was contrast-enhanced (Figure 1B). The mass was located posterior to the common carotid artery and mediosuperior to the subclavian artery (Figures 1C-1D). The mass appeared hypointense on T1-weighted magnetic resonance imaging (MRI) (Figure 2A), and gadolinium enhancement was observed only in a small area corresponding to the CT-enhancing portion (Figure 2B). The mass appeared hyperintense on T2-weighted images (Figure 2C). No tumor lesions within the orthotopic thyroid gland and no lymph node swelling were observed on CT and magnetic resonance imaging (MRI). Based on anatomical location and imaging features, a diagnosis of a mediastinal cystic lesion was made, with differential diagnoses including parathyroid, esophageal duplication, and bronchogenic cysts. We did not perform a fine-needle aspiration (FNA) because an FNA carries a risk of dissemination if a parathyroid cyst is suspected, especially if malignancy is possible.

**Figure 3:**
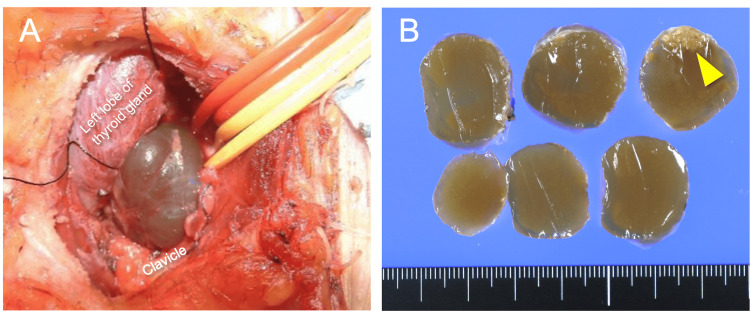
Intraoperative and resected specimen photograph (A) A cystic mass was identified below the thyroid gland. The common carotid artery was secured with the red vessel loop, and the vagus nerve was secured with the yellow vessel loop and pulled outward. (B) The resected specimen was a thin-walled cystic lesion with a smooth surface containing a gelatinous substance, showing papillary proliferation in a small area (arrowhead).

Transcervical tumor resection was performed to confirm the diagnosis and to remove the tumor. A collar-shaped incision was made in the anterior neck with neck extension. A cystic lesion was observed deep in the left common carotid artery (Figure 3A). The left recurrent laryngeal and vagus nerves were preserved, and the cystic mass was excised. The lesion showed no direct continuity with the thyroid gland. The resected specimen was a thin-walled cystic lesion with a smooth surface containing a gelatinous substance showing papillary proliferation in a small area (Figure 3B).

**Figure 4:**
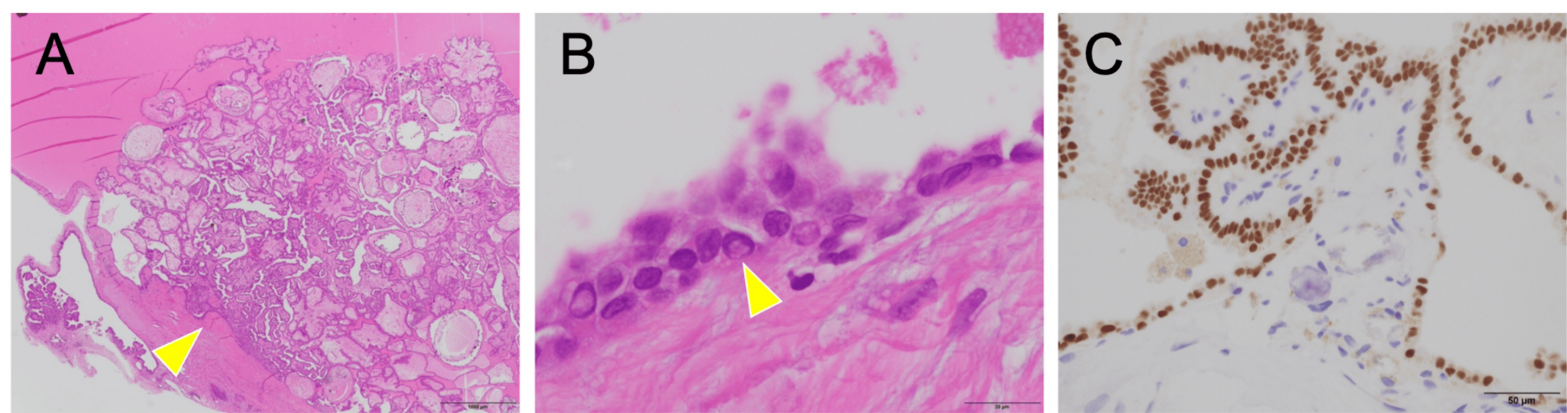
Pathological findings (A) Papillary proliferating cancer tissue (arrowhead) is observed (hematoxylin-eosin stain, ×20). (B) Intranuclear inclusions (arrowhead) are seen within the cancer cells (hematoxylin-eosin stain, ×1000). (C) Superficial epithelium stained positive for thyroid transcription factor-1 using immunochemistry (×400).

Pathological examination revealed that the inner surface of the cyst wall was lined with a columnar monolayer of epithelial cells. Atypical tumor cells and calcifications were observed in the small papillary area, but no normal lymphoid tissue was observed. (Figures 4A, 4B). Immunohistochemical staining revealed that the superficial epithelial cells were positive for thyroid transcription factor-1 and thyroglobulin, suggesting that the tumor originated from thyroid follicular cells. Based on these findings, the lesion was diagnosed as a papillary adenocarcinoma arising from an ectopic thyroid in the superior mediastinum.

## Discussion

The thyroid gland develops from the thyroid primordium, which originates from the cecal foramen of the tongue. Then, as the thyroglossal duct, it descends between the hyoid bone and the anterior surface of the thyroid cartilage, reaching its normal position. Consequently, ectopic thyroids are frequently found alongside the thyroglossal duct. However, ectopic thyroids are sometimes found outside of the thyroglossal duct. Therefore, ectopic thyroids can be classified into two types: dysgenesis and extragenesis. Dysgenetic-type ectopic thyroids result from the misplacement of the thyroid capsule into nearby tissues before formation [8]. In contrast, extragenetic ectopic thyroids occur in areas unrelated to thyroid development, with the orthotopic thyroid gland in its normal position. However, the reasons for this genesis are unclear [5]. Ectopic thyroids in the mediastinum, as in this case, account for only approximately 1% of all ectopic thyroids [9].

Ectopic thyroids consist of histologically normal thyroid tissue in 70%-90% of cases and are often asymptomatic [7, 10]. Most malignancies originate from the dysgenetic type ectopic thyroids, which are along the thyroglossal duct. Malignancies arising in other locations, particularly below the orthotopic thyroid gland, are rare [7, 11]. The current case is of thyroid carcinoma arising from an extragenetic ectopic thyroid located in the mediastinum, which is extremely rare.

The differential diagnosis of cystic mediastinal lesions can be divided by location, according to the International Thymic Malignancy Interest Group tricompartmental definition using cross-sectional CT imaging, such as the prevascular, visceral, and paravertebral compartments [12]. In the current case, the cyst was located in the visceral compartment, and the differential diagnoses included bronchogenic and esophageal duplication cysts [13, 14]. Although rare, parathyroid cysts have been reported [15]. Endoscopic ultrasound-guided FNA combined with cystic fluid analysis has been reported to be effective for the diagnosis of mediastinal cystic lesions [16]. However, diagnosing parathyroid cysts using FNA is difficult, and in malignant cases, there is a risk of easy dissemination to surrounding tissues. Therefore, FNA should be avoided in patients with suspected parathyroid cysts [15, 17]. In the current case, preoperative FNA was not performed because of the possibility of a mediastinal parathyroid cyst.

When thyroid cancer is detected outside the orthotopic site, it is important to differentiate whether it originates from an ectopic thyroid or is due to metastasis. Multiple masses are often observed in patients with thyroid cancer. Therefore, the absence of multiple masses, the absence of malignant tumors within the orthotopic thyroid gland, and the pathologic confirmation of normal thyroid tissue and cancer together indicate malignant tumors originating from an ectopic thyroid rather than a metastasis [18]. On the other hand, observing malignant tumors and normal lymphoid tissue together indicates lymph node metastasis [19]. In this case, the cancer was observed without normal thyroid or lymphoid tissue on pathological examination. Therefore, both ectopic thyroid cancer and lymphoid metastasis are possible. However, no tumor lesions were detected in the orthotopic thyroid gland on CT or MRI. Thus, we diagnosed the lesion as ectopic thyroid cancer rather than a metastatic disease. We did not perform an ultrasound examination or remove the orthotopic thyroid gland for pathological confirmation. Therefore, we cannot rule out the possibility that this case is a metastasis from a microcarcinoma. This is a limitation of the present report.

The treatment strategies for ectopic thyroid carcinoma remain controversial. For thyroglossal duct cyst carcinoma, which is representative of ectopic thyroid carcinoma, a few believe that total resection of the orthotopic thyroid is necessary after complete resection of the ectopic thyroid carcinoma [20, 21], while others believe that resection of the orthotopic thyroid is not necessary in cases where there are no obvious tumor lesions in the orthotopic thyroid to avoid postoperative thyroid and parathyroid dysfunction [4, 22]. In our case, the resection margin was pathologically negative, no obvious tumor lesions were detected in the orthotopic thyroid gland, and the postoperative thyroglobulin level was normal. Therefore, we considered that additional treatment, including radioactive iodine therapy, was unnecessary and preserved the thyroid gland. Although ectopic thyroid carcinoma is very rare, according to the guidelines for metastatic papillary carcinoma with an intact thyroid gland as seen on imaging, a total thyroidectomy with appropriate neck dissection might have been considered [23].

## Conclusions

We report a case of papillary carcinoma originating from an ectopic thyroid present as a cystic lesion in the superior mediastinum. Ectopic thyroid carcinoma arising below the normal thyroid gland, as in this case, is extremely rare. It should be included in the differential diagnosis of mediastinal cystic lesions. However, it must always be differentiated from microcarcinoma metastasis. Due to the rarity of ectopic thyroid carcinoma, follow-up and treatment strategies, including the necessity of total thyroidectomy, are controversial and require individualized consideration for each case.
